# What Can We Learn From a Human-Rights Based Approach to Disability for Public and Patient Involvement in Research?

**DOI:** 10.3389/fresc.2022.878231

**Published:** 2022-04-22

**Authors:** Jacqui Browne, Emma R. Dorris

**Affiliations:** ^1^Independent Researcher, Kerry, Ireland; ^2^PPI Ignite Network at UCD, University College Dublin Research Office, University College Dublin, Dublin, Ireland

**Keywords:** public and patient involvement, social model of disability, inclusive research, responsible research & innovation (RRI), engagement (involvement)

## Abstract

Public and Patient Involvement can align both the research process and its outcomes with the values, needs and expectations of society. By fostering the design of inclusive, engaged, and sustainable practices, research and research integrity can be improved. Devolving power to involve patients and relevant publics in deliberative decision making can produce better research outcomes. Disabled people are often categorized as “Hard to Reach.” There is a varied and complex ecosystem of societal challenges of living with a disability that reinforce this. However, if researchers are to meet their obligations under the UN Convention on the Rights of Persons with Disabilities, disabled people should be included in public and patient involvement for all research in which they have a stake. In this article we argue that a better understanding of rights-based approaches and the social model of disability within the wider research community can help to remove barriers to research involvement for disabled persons. We focus on articles 3, 4, and 9 of the Convention and discuss how the principles of participation, accessibility, and equality of opportunity can be applied to research involvement, and how their adoption can facilitate truly meaningful PPI in disability research.

## Introduction

“Nothing about us without us” is a phrase that dates to the devolution of power in the sixteenth century. In modern use, it is closely associated with the disability civil rights movement in the 1990s (1, 2) and the Convention on the Rights of Persons with Disabilities (CRPD), a major step toward addressing inequality (3, 4). The CRPD was the product of a multi-decade dialogue and a cumulation of building upon a series of legal documents that capture what is commonly called the human rights based approach to disability (5). The CRPD does not simply re-state existing rights. Rather it created a new rights framework and a new rights discourse to empower civil society and actively make human rights more obtainable for disabled people (6). It is perhaps unsurprising then that the powerful disability rights mantra of “Nothing about us without us” has been adopted by other advocacy movements, including the wider advocacy for public and patient involvement (PPI) in health and social care research.

Many of the transferable principles of inclusion highlighted in the CRPD are often not well-known in the research sphere beyond those working directly within civil and political rights or very specific areas of disability research. This even though the CRPD is an internationally legally binding instrument. Part of the reason for this is that the research system is often stuck within traditionalist concepts and systems (7). For example, the vulnerability concept in research on disabled people was traditionally designed to protect people (8). However, often disabled people are no more vulnerable than others. Rather, social contexts may place them in vulnerable positions more frequently (9). The effect of applying the vulnerability concept to disabled people in research inadvertently creates barriers for access to research and underrepresentation of disabled people in research (10). One of the goals underpinning PPI is to change the research ecosystem to be more inclusive so that research is more relevant, better, and has greater impact. A similar conceptual shift underlies the CRPD. Both echo movements away from traditionalist technocratic systems. There is much that can be learned from CRPD to advance our practice and policies in PPI.

Here, we will outline some key elements of the CRPD and discuss how they can be applied toward a more inclusive research involvement practice. We will examine specific parts of the CRPD and associated human rights-based approaches to disability and discuss how they can be applied to make PPI practice better and more inclusive. The CRPD provides a framework for a human rights-based approach; we explore how this may practically be implemented within the context of PPI in research.

## Models of Disability

### Medical Model of Disability

The medical model of disability arose from the biomedical model of healthcare. The biomedical model of healthcare dominated Western culture since the scientific revolution (11). This model places health as an absence of disease, and assumes disease to be fully accounted for by divergences from measurable biological variables (12). Thus, the biomedical model assumes all disease is independent of social behavior and environment. This model was originally designed as a scientific model to study the biology of disease. However, its narrow scope was adopted into medical practice and policy in the early 20th century (13, 14).

The medical model of disability views disability in terms of what is “wrong” with a person (15). The underlying assumption is that impairments or differences should be fixed or cured by medical treatments, even if there is no pain or illness associated with the impairment. One of the major issues of the medical model is that it is deterministic, implying that unless someone is “fixed” they are not, and cannot be, equal. The medical model is both reductionist and exclusionist and is widely rejected by disabled people.

### Social Model of Disability

The social model of disability views the social, civic, political, and economic environments as the disabling entity, not an individual's impairment. A social model perspective does not deny the reality of impairment nor its impact on the individual. However, it does challenge the physical, attitudinal, communication and social environment to accommodate impairment as an expected incident of human diversity. The social model frames “disability” as the result of the interaction between people living with impairments and an environment filled with physical, attitudinal, communication and social barriers.

Disabled people are not “objects” of charity, medical treatment, and social protection but “subjects” with rights, capable of claiming those rights, able to make decisions for their own lives based on their free and informed consent and be active members of society. The emphasis is on the physical, attitudinal, communication and social environment to change to enable people living with impairments to participate in society on an equal basis with others.

In line with the social model, we use the term “disabled person” throughout this article. We acknowledge that the CRPD and some others use the term of people or persons with disabilities.

### The Lagging Shift in Research

The United Nations Convention on the Rights of Persons with Disabilities (CRPD) marks the official shift toward the social model of disability as the internationally recognized way to view and address “disability”, in attitudes toward disabled people and approaches to disability concerns. Yet, Western law and policy is overwhelmingly based in a medical model of disability. This is often supplemented with charitable and supplementary measures to address need, rather than a rights-based approach (16). The adoption of the social model of disability across legislation, policy and decision-making processes is required to implement the CRPD.

Publicly funded research is not exempt from the requirements of the CRPD. Yet, engagement of disabled people in the decision making around research is still the exception, rather than the norm. For many years disabled people were categorized as “hard-to-reach” and indeed, often still are (17). The very use of this terminology provides an excuse for exclusion, as it implies the issue is “one within the group itself, not with your approach to them” -Smith 2006 (18). The adoption of the CRPD in 2006 outlined general principles yet now, 16 years later, we are still struggling with inclusion and PPI in research. These principles include non-discrimination, full and effective participation and inclusion in society, accessibility, and equality of opportunity. The reality is, however, that in PPI much work is still being done on the identification of barriers and development of special considerations to overcome them, rather than on the development of research ecosystems with universal design to facilitate natural inclusion (19).

## Learnings for PPI practice

### Article 4(3)

“*In the development and implementation of legislation and policies to implement the present Convention, and in other decision-making processes concerning issues relating to persons with disabilities, States Parties shall closely consult with and actively involve persons with disabilities, including children with disabilities, through their representative organizations*.”

Involvement is a key principle of the CRPD. Under the CRPD full and effective participation may include encouragement and receipt of appropriate support for participation in decision-making. For disabled PPI contributors to be free from stigma and feel safe and respected a number of factors should be considered. Full and effective participation is a process, not a one-time event. Values, such as respect, openness, reciprocity and flexibility identified by Ní Shé et al. (20) should be adopted to support inclusive, effective and collective PPI across all stages of involvement. Disabled people should be consulted to facilitate effective participation. Although inclusive PPI may incur greater financial costs, budgetary constraint is not a valid counter-argument to effective involvement of people with disabilities (21).

### Article 9 – Accessibility

“*To enable persons with disabilities to live independently and participate fully in all aspects of life, States Parties shall take appropriate measures to ensure to persons with disabilities access, on an equal basis with others, to the physical environment, to transportation, to information and communications, including information and communications technologies and systems, and to other facilities and services open or provided to the public, both in urban and in rural areas.”*

Under the social model of disability, society creates barriers that disable people living with impairments. In other words, disability is a social construct that results from the interaction between persons with impairments and barriers, both environmental and attitudinal, that hinder their full and effective participation. Thus, both disability and associated accessibility needs are evolving concepts. When focusing on creating an “accessible environment” for PPI, be dynamic in the approach. Include disabled people in any discussions or decisions about what their access needs are so that they can participate fully in the research process. Researchers will need to both manage the impact of an individual's impairment while also working to stop the research culture and ecosystem from creating barriers. As argued by Shakespeare, even if society removed barriers, people would be impacted by their impairments to varying extents (22). Be mindful that the act of creating an accessible environment can aid in minimizing the inconvenience of impairment, but this does not automatically equalize disabled people with those who are non-disabled (23). When considering access, take a broad approach with a focus on enabling capacity.

In the prospective design of PPI in research, a universal design approach is preferred. An in-depth discussion of universal design is beyond the scope of this manuscript, but is broadly defined as the design of environments and products to be usable by all people, to the greatest extent possible, without need for adaptation or specialized design (24). The concepts of universal design were also later applied to research design (25). Applying the broadest amount of universal design features facilitates the inclusion of the most diverse PPI contributors and should be applied where possible.

In the absence of universal design, Rios et al. pragmatically recommended a tiered approach of accommodations, and then modifications (26). Both accommodations and modifications are alterations to the standardized approach. Ideally, the standardized approach should not require special accommodations or modifications yet pragmatically, they are currently often required to enable full and equal involvement.

#### Physical Accessibility

Any meetings or events should be held in an environment with fully accessible buildings, roads, transportation, and other facilities, including digital facilities. Fully accessible generally means that people with disabilities can enter and/or make use of the environment with ease and without embarrassment. Frequently overlooked aspects include practical things such as signage and directions (are they available in braille or other alternative formats, are there audio and/or visual cues for guidance, are location maps at an accessible height, is signage easy to use/follow). Sensory impairments may require lighting, auditory, tactile specificities or sign language interpretation to create an enabling environment (27). There are a wide arrange of disabling environmental factors. A good working relationship with your PPI contributors and disability advocacy groups can greatly help in recognizing what is needed in your specific circumstance. Organizing your meetings in collaboration with a disabled person or consulting a disabled person on your facilities and plans, can greatly help in ensuring you meet your specific requirements for inclusive design.

#### Communication Accessibility

Universally for PPI, researchers should ensure that the language used is always appropriate and acceptable. In the area of disability, there are numerous resources on appropriate language use. For example, in Ireland, the National Disability Authority produced guidelines for effective consultation with disabled people (2002) and are in the process of updating these guidelines in consultation with both DPOs and with input from a broad range of individuals with disabilities (28). Researchers should make themselves familiar with language and terminology considerations both with existing guidelines, and through effective, collaborative working relationships with disabled people and DPOs.

Communication is not just about language use. Communication accessibility recognizes that people have different communication needs. There is a wide array of local, national, and international standards that govern accessible communications. Yet the level of effective implementation of communication accessibility remains low (29, 30). Communication is paramount in PPI, and accessible communication should be a priority for any researcher engaging in it. Accessible communication seldom just happens. To enable it to occur dedicated effort is required to identify and provide adequate resources and work toward solutions (31). It should be thoroughly planned and resourced as appropriate to facilitate effective communication that allows for freedom of thought and expression to be exercised. What needs to be done to ensure communication accessibility will vary by context, but adoption of universal design concepts for communication should be a starting point (25, 32, 33). The degree of additional accommodations or modifications will vary widely. It may be that accommodations are required for persons with significant speech and physical impairments, sensory impairments, or to minimize fatigue or discomfort. The important aspect is to collaborate with the disabled people and DPOs that you are involving so that the issues or potential issues can be identified, and solutions implemented to enable full participation of all involved.

#### Information Accessibility

Similarly to communications, there often remains a lag in implementation of effective information accessibility despite a wide variety of policies and instruments governing this area (30). If information is not accessible, it excludes people from full and effective participation. For information to be accessible, it should be provided in a format that allows all users to equally access the content. In PPI, effective and collaborative planning with disabled people and DPOs can help to identify what information formats will be needed for each research project.

Making information accessible can include avoiding the use of jargon and providing “plain English” versions of documents. It may mean the use of direct language to enable people to comprehend the information being provided. It may also mean the use of Information and Communication Technology (ICT) to create accessible materials in text, audio, and video to facilitate information accessibility. There are a wide array of guidance documents to assist with information accessibility, including the ICT for information accessibility in learning (ICT4IAL), guidelines for accessible information, available in multiple languages (34).

#### Attitudinal Accessibility

A major stumbling block to meaningful and sustainable involvement of disabled people in PPI is a perceived lack of respect. In the literature, this may be described as process issues, professional behavior, or sensitivity (20, 35, 36). Disabled people should have equal access to involvement in a manner that respects their dignity. Including a disabled person as a token instrument does not create an enabling environment.

Often, the perceptions of individuals' capacity for involvement may diverge considerably from their actual capacity. There can be deep-rooted social-structural negative perceptions of the capacity of disabled people. The act of inviting someone to become involved does not automatically negate these perceptions. In addition to the socially embedded perceptions often inherent in researchers and research organizations, disabled people themselves can lack self-belief that they can contribute successfully and meaningfully (37–40). The mechanisms underlying these attitudes are complex and multidimensional. However, acting as if they do not exist is counterproductive. Underlying biases and discrimination against disabled people do exist (41). There should be dedicated effort on the part of any researcher or research organization engaging in PPI to acknowledge this, and then to work to identify and create solutions to enable a truly respectful collaboration.

### Article 3(e)- Equality of Opportunity

“*Equalization of opportunities, as a general principle of the Convention under article 3, marks a significant development from a formal model of equality to a substantive model of equality. Substantive equality [….] seeks to address structural and indirect discrimination and takes into account power relations. It acknowledges that the “dilemma of difference” entails both ignoring and acknowledging differences among human beings in order to achieve equality.”*

#### Inclusion of Disabled People in All Health and Social Care Research

Much of the discussion in this paper has been framed in the context of disability research. It is important to note, that the recommendations for learnings are applicable across all health and social care research disciplines. Disabled people have a wide range of health conditions, just as the general population does. Yet disabled people are underrepresented in mainstream health research (25, 42). Disabled people experience health care disparities largely due to multifaceted social disadvantages (43). An estimated quarter of working age adults living with a chronic condition also have a disability, whereas up to 90% of those with a disability also had a chronic condition (44). Thus, disabled people will typically be part of the population affected by non-disability specific health issues. It is therefore important to ensure that all research involvement is open, enabling, and inclusive to disabled people.

#### General Comment 7-Distinction Between Organizations of Persons With Disabilities and Other Civil Society Organizations

*13.Organizations of persons with disabilities should be distinguished from organizations “for” persons with disabilities, which provide services and/or advocate on behalf of persons with disabilities, which, in practice, may result in a conflict of interests in which such organizations prioritize their purpose as private entities over the rights of persons with disabilities. States parties should give particular importance to the views of persons with disabilities, through their representative organizations, support the capacity and empowerment of such organizations and ensure that priority is given to ascertaining their views in decision-making processes*.

The distinction between Disabled Persons Organizations (DPOs) from organizations “for” disabled people is an important one. DPOs are led, directed, and governed by disabled persons and their majority membership are disabled persons. These organizations are distinct from other organizations led by health professionals, family and others run on behalf of disabled people. Disabled people have better knowledge of their needs, priorities and experiences and are therefore in a better position to provide a voice and knowledge of their needs. Often, organizations “for” disabled people follow a medical model of disability whereas DPOs follow the social model (45). When engaging with an organization for research involvement you should be clear and transparent about who you are involving. Disabled people consider that part of the problem and invisibility they have experienced over the years is because service providers, amongst others, have represented them or advocated for their needs, rather than being given the opportunity to participate themselves directly (46). Research involvement should facilitate the inclusion of disabled people directly to ensure true benefit from the knowledge, skills, and experience of disabled people.

## Conclusions

The CRPD is a powerful international human rights treaty that has resulted in a dedicated focus on changing public attitudes and responsibility for the inclusion of disabled people in all aspects of society. The explicit focus on equality and participation in the CRPD reflects some of the goals underpinning PPI and the movement away from the narrow biomedical model to a wider social model. Researchers should clearly identify and give priority to supporting the capacity and participation of disabled people and DPOs in their research. The rise of PPI in health and social care research has opened researchers to new and novel views on both their research and their approaches to it. Breaking out of the traditional health research modes can be difficult for some, especially if the research environment does not foster these changes. Research institutions must move beyond policies on inclusion and ensure implementation of fully inclusive environments. This includes adequate resourcing to facilitate active and on-going involvement. Inclusive budgeting of research should become the norm. DPOs, research institutes and research policy makers should work in close consultation to develop inclusive norms that foster accessibility and non-discrimination across research practices. The CRPD rights-based approach can be a useful instrument to promote these changes. There is a wealth of learnings, guidance and reflections from the application and implementation of the CRPD that can be useful for better implementing inclusive research involvement. There is much guidance available, learnings from the CRPD and rights-based approach should now be implemented as a matter of standard practice in PPI.

## Data Availability Statement

Publicly available datasets were analyzed in this study. This data can be found here: https://www.un.org/development/desa/disabilities/convention-on-the-rights-of-persons-with-disabilities.html.

## Author Contributions

JB is a disabled person, advocate and patient partner. ED is a PPI specialist with a background in translational research. All authors listed have made a substantial, direct, and intellectual contribution to the work and approved it for publication.

## Funding

ED was funded under the PPI Ignite Network (Grant number PPI-2021-001), with funding provided by the Health Research Board, Irish Research Council and University College Dublin.

## Conflict of Interest

The authors declare that the research was conducted in the absence of any commercial or financial relationships that could be construed as a potential conflict of interest.

## Publisher's Note

All claims expressed in this article are solely those of the authors and do not necessarily represent those of their affiliated organizations, or those of the publisher, the editors and the reviewers. Any product that may be evaluated in this article, or claim that may be made by its manufacturer, is not guaranteed or endorsed by the publisher.
